# Does Gait with an Ankle Foot Orthosis Improve or Compromise Minimum Foot Clearance?

**DOI:** 10.3390/s21238089

**Published:** 2021-12-03

**Authors:** Pedro Fonseca, Leandro Machado, Manoela Vieira Sousa, Ricardo Sebastião, Filipa Sousa, Joana Figueiredo, Cristina P. Santos, João Paulo Vilas-Boas

**Affiliations:** 1 Porto Biomechanics Laboratory (LABIOMEP), University of Porto, 4200-450 Porto, Portugal; pedro.labiomep@fade.up.pt (P.F.); manoelavsousa@fade.up.pt (M.V.S.); rsebastiao@fade.up.pt (R.S.); filipas@fade.up.pt (F.S.); jpvb@fade.up.pt (J.P.V.-B.); 2 Center of Research, Education, Innovation and Intervention in Sport (CIFI2D), Faculty of Sports of the University of Porto, 4200-450 Porto, Portugal; 3 Center for MicroElectroMechanical Systems (CMEMS), University of Minho, 4800-058 Guimarães, Portugal; joana.figueiredo@dei.uminho.pt (J.F.); cristina@dei.uminho.pt (C.P.S.)

**Keywords:** gait analysis, minimum foot clearance, orthosis, rehabilitation, compensation, swing phase

## Abstract

The purpose of this study was to investigate if the use of an ankle foot orthosis in passive mode (without actuation) could modify minimum foot clearance, and if there are any compensatory mechanisms to enable these changes during treadmill gait at a constant speed. Eight participants walked on an instrumented treadmill without and with an ankle foot orthosis on the dominant limb at speeds of 0.8, 1.2, and 1.6 km/h. For each gait cycle, the minimum foot clearance and some gait linear kinematic parameters were calculated by an inertial motion capture system. Additionally, maximum hip and knee flexion and maximum ankle plantar flexion were calculated. There were no significant differences in the minimum foot clearance between gait conditions and lower limbs. However, differences were found in the swing, stance and step times between gait conditions, as well as between limbs during gait with orthosis (*p* < 0.05). An increase in hip flexion during gait with orthosis was observed for all speeds, and different ankle ranges of motion were observed according to speed (*p* < 0.05). Thus, the use of an ankle foot orthosis in passive mode does not significantly hinder minimum foot clearance, but can change gait linear and angular parameters in non-pathological individuals.

## 1. Introduction

Human locomotion on land is performed mainly by walking in an erect position, which is enabled by the successive advance of the lower limbs, while keeping a dynamic control of balance. This coordination ability reflects a complex combination of actions of the musculoskeletal system [1,2], which may be impaired by age-related degeneration or pathological alterations [3].

Motor disabilities resulting from pathologies, such as stroke, palsy or multiple sclerosis, may cause a significant toll on an individual’s ability to walk and their daily living. These difficulties may result in falls [4], which may lead to additional side effects, with the potential to increase motor impairment. In older adults, tripping is a leading cause of accidental falls [5,6], and although many causes may be related to fall occurrence, the minimum foot clearance (MFC) has been associated with an increased risk of falling [2,7]. MFC is defined as the minimum vertical distance between the ground and the foot during the swing phase [8], and changes in ankle dorsiflexion considerably influence the MFC in gait [9].

Ankle foot orthoses (AFOs) are devices that can be used to provide additional support to ankle movement, either in a passive or active manner. Tyson et al.’s [10] review has shown that the use of AFOs can prevent foot-drop, facilitate weight-bearing on a paretic leg and reduce the energy cost of walking. Therefore, AFO use can be important for several lower limb pathologies, effectively changing gait patterns. For example, increased ankle dorsiflexion at heel strike and swing phase has been reported [11], although without a corresponding change in the hip flexion pattern in post-stroke gait [11,12]. Another compensatory mechanism is limb shortening, where the distance from the hip to the toe is reduced due to increased flexion of the hip and knee. Pongpipatpaiboon et al. [13] observed that toe clearance is increased with AFO use due to limb shortening during the swing phase in hemiparetic gait. Matsuda et al. [14] observed a similar behavior, with limb shortening and hip elevation as a trade-off for achieving ground clearance in hemiparetic patients. Greene and Granat [15] also observed that in paraplegic gait with AFO, knee flexion is not enough to maintain gait without compensatory movements, with it also being necessary to perform increased ankle dorsiflexion to achieve an adequate ground clearance. Therefore, gait while using AFO may show alterations in MFC, but also compensatory movements, to maintain functional clearance.

The purpose of this study was to investigate if the use of an AFO can modify the MFC during treadmill gait at constant slow speed in healthy young adults. Additionally, we aimed to identify which are the compensatory mechanisms that enable these changes by assessing the lower limbs’ linear and angular kinematics. Therefore, two hypotheses were established and analyzed: (1) orthotic gait does not significantly affect the MFC at low gait speeds and (2) compensatory movements are developed to allow a lower limb with an orthosis to maintain an MFC identical to the contralateral limb.

## 2. Materials and Methods

### 2.1. Participants

A group of eight participants (4 female, 4 male), all reporting right limb dominance, volunteered to participate in this study. The group showed an average age of 24.3 ± 1.8 years old (male: 24.0 ± 1.2; female: 24.5 ± 2.4), 171.0 ± 7.7 cm height (male: 175.5 ± 6.7; female: 166.3 ± 5.7), 68.3 ± 10.1 kg of body mass (male: 77.0 ± 2.9; female: 59.5 ± 5.0) and a body mass index of 23.3 ± 2.9 kg/m^2^ (male: 25.0 ± 2.6; female: 21.6 ± 2.3). All participants were healthy without reporting any known locomotion or balance impairment, and they had not suffered any musculoskeletal injury in the previous six months.

All participants were informed of the study’s objectives and methodology, and were provided with an informed consent form, which they read and signed. The study was approved by the University of Minho Research in Life and Health Sciences Ethics Committee, with the protocol number CEICVS 006/2020.

### 2.2. Experimental Setup

Two experimental gait conditions were analyzed: (i) unconstrained gait with the participants walking without any AFO or other assistive devices; (ii) gait with an AFO attached to the participant’s dominant limb—the right lower limb (RL)—while leaving the contralateral limb—the left lower limb (LL)—free.

The AFO used was adapted from the H2-Exoskeleton (Technaid S.L., Madrid, Spain) and weighed 2.1 kg. The orthosis provided a single degree of freedom on the sagittal plane and was configured with zero-impedance control, meaning that the orthosis allowed ankle dorsal and plantar flexion, but did not provide any assistance to that movement, thus acting in passive mode. The limb without the orthosis was shod with an identical shim to that of the limb with the orthosis in order to avoid lower limb length differences.

Motion data was recorded with an MTw Awinda motion capture system (Xsens Technologies B.V., Enschede, The Netherlands) operating at a 100 Hz sampling frequency. Seven inertial measurement units were placed and secured to the lower limbs with the provided fixation straps. The sensors were placed on the posterior region of the pelvis, on the medial aspect of the right and left thighs and shanks, and on the dorsal aspect of the feet. The sensors’ position on the thigh and shank differed from the manufacturer recommendation (lateral positioning) to avoid any mechanical interference of the AFO over the inertial units. The same sensor positioning was kept for the condition without the AFO to ensure comparable results.

**Figure 1:**
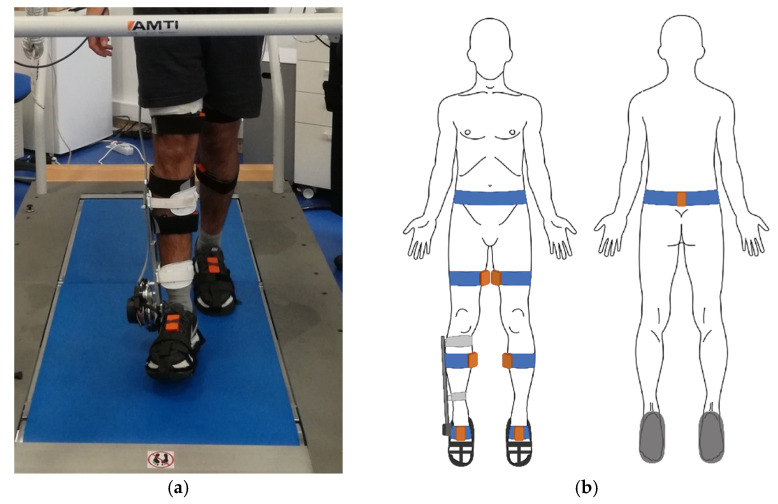
Representation of the (**a**) experimental setup with the participant walking on the instrumented treadmill while wearing the inertial motion capture system in the lower limbs, along with the foot ankle orthosis in the right lower limb and (**b**) inertial sensor placement locations in the participant’s lower limbs.

All experimental procedures were performed on an instrumented treadmill (AMTI Inc., Watertown, MA, USA) to allow the participants to walk at a constant speed. A 6-min familiarization walk on the treadmill at 1.2 km/h speed was performed before data collection [16]. Next, a 6-min trial was performed for each of the following speeds: 0.8, 1.2, and 1.6 km/h. These very slow gait speeds were selected because the AFO will be used in an impaired population, and these correspond to the range enabled by the H2-Exoskeleton AFO [17]. A representation of the experimental setup can be seen in Figure 1.

### 2.3. Data Analysis

The collected data were processed with Visual3D v2021.03.2 (C-Motion, Germantown, MD, USA), using the built-in XSens biomechanical model. A central 5-min period was selected for analysis, and the gait events of heel strike (HS) and toe off (TO) were identified for each lower limb. For a given lower limb, the gait cycle was established as the period between consecutive HS, the stance phase as the period between HS and TO, and the swing phase as the period between TO and HS. These events were used to calculate gait linear kinematic parameters, namely: step and stride lengths, step time, and swing and stance times. All temporal parameters were normalized to the duration of the corresponding limb gait cycle duration, while the step length were normalized to the participant’s stature.

The joint angles of the hip, knee, and ankle, as well as their range of motion (ROM), were calculated for the sagittal plane during the swing phase. We retained for further analysis the peak flexion from the hip and knee and the peak plantar flexion from the ankle.

Foot clearance was calculated, for each foot, as the vertical displacement of the distal portion of the foot (DPF) in relation to the ground. Minimum foot clearance (MFC) was calculated as the difference between the DPF height during the heel’s maximum height, and the minimum DPF between TO and HS.

### 2.4. Statistical Procedures

Data normality was assessed with the Kolmogorov–Smirnov test, and results were described as mean and standard deviation. Then, a repeated measures MANOVA was applied to test the effects between gait conditions (without AFO and with AFO), speed (0.8, 1.2 and 1.6 km/h) and limbs (RL and LL). When main effects were found, a Bonferroni post hoc test was conducted. The G * Power 3.1.7 software (University of Kiel, Kiel, Germany) was used to calculate the effect size and to determine the power of the analysis using Cohen’s *d* interpretation (small: >0.2; moderate: >0.50; large: >0.80) [18]. All statistical procedures were performed on SPSS version 27 (IBM, Armonk, NY, USA) with a significance level of α = 0.05.

## 3. Results

### 3.1. Minimum Foot Clearance

No significant differences were found in terms of MFC between gait conditions, or limbs, in all gait speeds tested, as presented in Table 1.

When comparing gait conditions, speed, and limb—in terms of MFC—no interaction was found (gait * limb * speed: *F* = 0.23, *p* = 0.68, *d* = 0.13). Furthermore, no change was observed for gait * speed (*F* = 1.99, *p* = 0.15, *d* = 0.37) or gait * limb (*F* = 2.38, *p* = 0.14, *d* = 0.41).

### 3.2. Gait Linear Kinematic Parameters

When gait linear kinematic parameters were compared in terms of gait condition, speed, and limb (gait * speed * limb), no interaction was found. However, an interaction between gait condition and limb (gait * limb) was found for the stance (*F* = 16.10, *p* < 0.01, *d* = 1.07), swing (*F* = 15.04, *p* = 0.002, *d* = 1.03), and step (*F* = 26.06, *p* < 0.01, *d* = 1.36) times, as well as for step length (*F* = 7.27, *p* = 0.01, *d* = 0.72). This indicates that, while no differences between limbs were found in gait without AFO for these parameters, differences between limbs do exist in gait with AFO. In this gait condition, the right lower limb (RL) shows a lower stance time (*d* = 1.98), increased swing (*d* = 1.63) and step (*d* = 4.66) times. When comparing the same limb between gait conditions, the RL shows a decrease in stance (*d* = 2.05) and increase in swing (*d* = 1.63) times in gait with AFO. Still comparing gait conditions, and during gait with AFO, the RL shows an increase in step time (*d* = 1.96), while the left lower limb (LL) shows a decrease in step time (*d* = 2.38). Finally, the RL step length was found to be increased in gait with AFO (*d* = 1.91).

As previously mentioned, no differences were found between the speeds of the RL and the LL for any given gait condition. However, a significant interaction between gait condition and speed (gait * speed) was found for step (*F* = 11.29, *p* < 0.01, *d* = 0.89) and stride (*F* = 19.90, *p* < 0.01, *d* = 0.99) lengths. This indicates a difference in these parameters between gait conditions, independently of the limb, at 1.2 (step: *d* = 1.21; stride: *d* = 1.85) and 1.6 km/h (step: *d* = 1.21; stride: *d* = 1.66), but not at 0.8 km/h. Further comparisons have revealed that step and stride lengths in gait without orthosis differ for 0.8 vs. 1.2 km/h (step: *d* = 0.25; stride: *d* = 0.78), 0.8 vs. 1.6 km/h (step: *d* = 0.25; stride: *d* = 0.91), while in gait with orthosis these differences occur for 0.8 vs. 1.2 km/h (step: *d* = 0.64; stride: *d* = 5.12) and 0.8 vs. 1.6 km/h (step: *d* = 1.06; stride: *d* = 0.73), and for 1.2 vs. 1.6 km/h (stride: *d* = 1.17).

The gait linear kinematic parameters and respective comparisons are presented in Table 2.

### 3.3. Joint Kinematic Parameters

In terms of joint kinematics, the interaction between gait condition, limb, and speed (gait * limb * speed) only revealed differences at the ankle ROM (*F* = 4.03; *p* = 0.03; *d* = 0.53). These are related to differences between gait speeds, namely, a ROM decrease at 1.2 vs. 1.6 km/h (*d* = 0.44) for the right lower limb (RL) during gait without orthosis. On the contrary, during gait with orthosis, only the LL shows differences, including a ROM increase for 0.8 vs. 1.2 km/h (*d* = 0.85) and 0.8 vs. 1.6 km/h (*d* = 0.89).

When comparing gait condition and limb (gait * limb), a significant interaction was found for hip angle (*F* = 6.95, *p* = 0.02, *d* = 1.03), indicating an angle increase of the RL from gait without to gait with orthosis (*d* = 1.06), and significantly different from the LL (*d* = 0.42) in the latter condition.

The interaction between gait condition and speed (gait * speed) shows significant differences at the ankle angle (*F* = 8.10, *p* = 0.003, *d* = 0.76), hip ROM (*F* = 6.85, *p* = 0.005, *d* = 0.70) and ankle ROM (*F* = 6.06, *p* = 0.007, *d* = 0.65). Hip ROM differs between gait conditions at 1.2 (*d* = 0.52) and 1.6 km/h (*d* = 0.90). Comparing these parameters in terms of speed between gait conditions, differences are found for 0.8 vs. 1.2 km/h (ankle: *d* = 0.69, hip ROM: *d* = 0.28) and 0.8 vs. 1.6 km/h (ankle: *d* = 0.69, hip ROM: *d* = 0.31, ankle ROM: *d* = 0.96) in gait with orthosis.

The comparison of joint angles and range of motion between gait conditions, for each speed and limb, is presented in Table 3.

## 4. Discussion

The AFO used in this study was kept on a passive setting, although it was designed to operate as an active assistive device, supporting and enabling dorsal and plantar flexion of the ankle during gait. This implies that, for the purposes of this study, the assistive motor did not produce any flexor or extensor torques. This was done so that the effect of adding such device on a lower limb, causing an increase in mass and having the potential to change the right lower limb (RL) proprioception and kinematics, could be analysed. In fact, Wang et al. [19] reported that treadmill walking while carrying an asymmetrical load may change lower limb coordination, while Mattes et al. [20] and Lin-Chan et al. [21] reported a potential to change gait performance and its metabolic cost. This study aimed to demonstrate, on a healthy sample of participants, the inherent effects of orthosis use during gait. It should also be mentioned that the gait speeds presented here are considerably lower than the normal healthy standard, but are in accordance with the lower speeds observed in pathological gait while using AFO [13,22].

During the analysed gait speeds, there was not a significant MFC difference between the right (RL) and left (LL) lower limbs, although the latter was associated with a higher mean. However, this was observed in both gait without and with AFO, and therefore is likely to reflect the effect of right-limb (RL) dominance of the sample enrolled in this study. This is not in agreement with the results of Nagano et al. [23] for gait without AFO, who reported a higher MFC for non-dominant limbs. These authors also reported that during treadmill gait, the MFC was lower than that observed during over-ground gait, which was also not observed in this study. Indeed, the MFC values of the RL are within the upper tier of the range of 0.45–3.5 cm observed in young and elderly individuals in diverse gait conditions [8,13,24,25,26,27]. Our first hypothesis stated that the AFO would not affect MFC at low speeds, and indeed results seem to support this claim, as no differences between limbs and gait conditions were found.

The studied gait linear kinematic parameters showed several differences between limbs and gait conditions, which are particularly relevant in the temporal domain. While the gait without AFO did not show any difference between limbs, gait with AFO differs from this reference condition. When walking with an orthosis on the dominant limb, a reduction of stance time, and a concomitant increase of swing and step times of the RL occurs. The step length of the RL in gait with orthosis was not statistically different from the LL, although showing a higher mean for all velocities. In fact, the meta-analysis of Choo and Chang [28] has demonstrated that the use of an AFO tends to favor step length increase and, in our case, is enough to constitute a difference to the RL when not using AFO. This seems to indicate a gait pattern where the AFO user will transition more rapidly from stance to swing phase, and use the additional swing time to control the RL forward advance, leading to a longer step time. This increase in time may also explain the apparently higher MFC, as a higher clearance may be possible with a longer step time. Therefore, we may conclude that the increased swing and step times may be related to a slower limb translation rather than achieving significantly higher distances with the RL, or may even be related to the counteracting of some instability caused by the AFO [29]. This agrees with Vistamehr et al. [30], who observed a decrease in the propulsive impulse in the AFO limb compared to the contralateral leg. The fact that differences in step and stride length exist between speeds in both gait conditions also seems to agree with the systematic review of Fukuchi et al. [31], who reported that young adults tend to have short step lengths at slower speeds. In fact, both the RL and LL, without and with AFO in this study, showed lower step and stride lengths at lower gait speeds.

In this study, we chose to analyze the maximum flexion of the hip and knee, while for the ankle, only maximum plantar flexion was retrieved. This was done to understand if increased hip and knee flexion was improving clearance, and to what extent the ankle was in plantar flexion during the swing phase. Mechanisms associated with a strategy of limb shortening, where increased flexion of the lower limb allows the overall reduction of the distance from the hip to the toe, was observed in gait with AFO. Although this increased flexion was expected to occur at the knee [28], with an increased joint range of motion [13], only the hip angle of the RL showed increased flexion. This was not observed in gait without AFO, thus indicating an alteration associated with the use of an AFO. This can indicate the use of hip flexion as an aid for the elevation of the RL which, due to the AFO, shows increased mass, and results in the previously mentioned alterations in gait linear kinematic parameters. At the ankle, differences were found at the ROM within, but not between, gait conditions—nor between limbs. Considering these differences between the RL and LL, our hypothesis that the AFO would induce compensatory movements on the RL is supported, with the notion that this is promoted by hip control rather than the ankle’s.

## 5. Limitations

As the conclusions drawn from this study are a direct result of the methodological approach, some limitations should be mentioned. One is certainly the use of an inertial motion capture system. Although the inertial measurement sensors have been validated, particularly in the sagittal plane [32], and have been reported as reliable [33], the measurement of foot clearance may be more accurate if performed with optical motion capture systems [34]. The latter method allows the placement of tracking markers in the exact position of the heel and toe, and has a sub millimetric spatial resolution. While the inertial approach still allows reliable and reproducible data collection, it also limits the analysis of other compensatory movements such as hip elevation and limb shortening calculation.

Another limitation is related to the use of an AFO in healthy participants in a passive mode. While this is not the intended use of an AFO, the results present evidence that this device, probably due to increased mass, does promote differences in gait linear and joint kinematic parameters, although they do not show significant changes in MFC. These results should not be generalized, and caution is advised when analyzing pathological samples. However, by using a healthy sample, it was possible to observe that MFC measurement is not enough for the validation of an AFO’s ability to support the foot during gait, and should be analyzed along with, at the least, other gait linear or joint kinematic parameters.

## 6. Conclusions

The use of an AFO on passive mode does not significantly hinder MFC on the dominant lower limb in healthy young individuals at low gait speeds. However, it may change the temporal distribution of the stance, swing, and step times of both limbs, as well as the hip flexion. These gait alterations support the notion that MFC should be analyzed in conjugation with gait linear or joint kinematics to truly identify how the AFO restores lower limb function.

Further studies should focus on the analysis of the effect of the AFO’s total mass and its distribution. It is also recommended that higher speeds be tested to analyze if these results remain valid.

## Figures and Tables

**Table 1:** Minimum foot clearance (MFC) of the right (RL) and left (LL) lower limbs without and with orthosis, for each gait speed, expressed as mean (standard deviation).

Gait Condition	Gait Speed	Minimum Foot Clearance (cm)	
		Right Lower Limb (RL)	Left Lower Limb (LL)
Without AFO	0.8 km/h	3.39 (2.14)	3.49 (1.70)
	1.2 km/h	2.92 (1.19)	3.05 (1.04)
	1.6 km/h	2.89 (1.49)	3.03 (1.03)
With AFO	0.8 km/h	3.27 (1.52)	4.49 (1.29)
	1.2 km/h	3.90 (1.85)	4.77 (2.02)
	1.6 km/h	3.37 (1.48)	4.53 (2.25)

**Table 2:** Gait linear kinematic parameters of the right (RL) and left (RL) lower limbs without and with orthosis, for each gait speed, expressed as mean (standard deviation).

Gait Condition	Limb	Gait Speed	Duration (% Gait Cycle)			Length (% Stature)	
			Stance	Swing	Step	Step	Stride
Without AFO	Right (RL)	0.8 km/h	77.10 (3.59)	23.15 (3.94)	51.13 (2.57)	21.01 (5.06)	45.65 (8.72)
		1.2 km/h	73.08 (3.14)	26.91 (3.14)	51.38 (1.76)	23.64 (4.41)	51.06 (5.99)
		1.6 km/h	69.52 (1.86)	30.47 (1.86)	51.55 (1.80)	24.72 (4.46)	52.80 (7.54)
	Left (LL)	0.8 km/h	77.46 (2.69)	22.55 (2.70)	49.17 (0.44)	24.52 (4.32)	45.66 (8.66)
		1.2 km/h	73.57 (2.16)	26.46 (2.16)	49.00 (1.72)	27.13 (2.68)	51.06 (5.98)
		1.6 km/h	69.96 (0.76)	30.03 (0.76)	48.91 (1.57)	27.73 (3.64)	53.09 (7.86)
With AFO	Right (RL)	0.8 km/h	72.48 (2.65) *	27.51 (2.65) *	56.80 (4.48) *	24.46 (2.50)	47.65 (2.21)
		1.2 km/h	67.76 (3.24) *	32.23 (3.24) *	58.92 (3.19) *	31.12 (3.11)	59.21 (2.63)
		1.6 km/h	64.94 (3.96) *	35.05 (3.96) *	56.60 (1.71) *	32.03 (2.73)	62.25 (2.60)
	Left (LL)	0.8 km/h	77.16 (2.31)	22.83 (2.31)	43.31 (4.47)	22.87 (3.10)	47.50 (1.98)
		1.2 km/h	73.06 (3.09)	26.93 (3.09)	41.12 (3.16)	27.35 (3.61)	59.29 (2.57)
		1.6 km/h	69.95 (2.58)	30.40 (2.58)	43.44 (1.69)	29.21 (2.87)	62.26 (2.57)

**Note:** The gray color indicates a significant difference between gait conditions for the same limb (*p* < 0.05); an asterisk (*) indicates significant difference between limbs during a given gait condition (*p* < 0.05).

**Table 3:** Maximum angle and range of motion of the right (RL) and left (LL) lower limbs without and with orthosis, for each gait speed, expressed as mean (standard deviation).

Gait Condition	Limb	Gait Speed	Joint Angle (Degrees)			Joint Range of Motion (Degrees)		
			Hip	Knee	Ankle	Hip	Knee	Ankle
Without AFO	Right (RL)	0.8 km/h	26.86 (5.23)	55.51 (10.16)	−12.55 (4.65)	27.69 (8.37)	47.24 (12.18)	22.54 (8.42)
		1.2 km/h	25.61 (5.77)	56.55 (7.40)	−13.23 (5.82)	28.95 (7.72)	50.41 (8.77)	23.68 (7.90) ^c^
		1.6 km/h	25.46 (7.57)	58.31 (8.95)	−10.11 (7.71)	27.77 (8.08)	53.37 (7.34)	19.24 (7.13) ^b^
	Left (LL)	0.8 km/h	28.71 (2.85)	57.05 (8.35)	−12.67 (3.83)	29.30 (8.39)	46.06 (11.87)	20.28 (9.27)
		1.2 km/h	27.15 (3.08)	56.95 (6.77)	−13.29 (4.85)	30.38 (6.84)	49.61 (8.50)	20.65 (7.74)
		1.6 km/h	27.09 (5.88)	59.14 (7.29)	−11.78 (4.89)	29.06 (7.82)	51.66 (8.08)	18.40 (8.17)
With AFO	Right (RL)	0.8 km/h	31.79 (6.79) *	62.42 (4.96)	−14.00 (4.02)	29.85 (3.61)	51.02 (8.43)	16.33 (2.73)
		1.2 km/h	34.55 (7.83) *	61.92 (7.16)	−16.72 (2.85)	36.97 (6.97)	52.42(10.36)	19.95 (3.73)
		1.6 km/h	33.79 (8.26) *	62.87 (6.90)	−15.52 (3.53)	38.37 (8.17)	54.48 (8.99)	18.43 (3.25)
	Left (LL)	0.8 km/h	29.57 (4.94)	61.19 (6.16)	−6.70 (5.38)	33.81 (4.69)	54.13 (9.23)	16.63 (3.64) ^b,c^
		1.2 km/h	30.73 (5.19)	60.31 (7.85)	−12.14 (7.02)	38.88 (6.75)	54.52 (11.62)	22.55 (4.40) ^a^
		1.6 km/h	31.27 (5.84)	61.69 (8.13)	−11.81 (6.01)	40.00 (7.63)	56.55 (9.12)	22.08 (4.24) ^a^

**Note:** The gray color indicates a significant difference between gait conditions for the same limb (*p* < 0.05); an asterisk (*) indicates a significant difference between limbs in a given gait condition (*p* < 0.05); difference at ^a^ 0.8, ^b^ 1.2 and ^c^ 1.6 km/h for the same limb and gait condition.

## Data Availability

The data underlying this study is available from the corresponding author upon request.
